# µ-Crystallin Is Associated with Disease Outcome in Head and Neck Squamous Cell Carcinoma

**DOI:** 10.3390/jpm11121330

**Published:** 2021-12-08

**Authors:** Bernhard J. Jank, Markus Haas, Julia Schnoell, Michaela Schlederer, Gregor Heiduschka, Lukas Kenner, Lorenz Kadletz-Wanke

**Affiliations:** 1 Department of Otorhinolaryngology and Head and Neck Surgery, Medical University of Vienna, A-1090 Vienna, Austria; bernhard.jank@meduniwien.ac.at (B.J.J.); markus.haas01@icloud.com (M.H.); julia.schnoell@meduniwien.ac.at (J.S.); gregor.heiduschka@meduniwien.ac.at (G.H.); 2 The Division of Experimental Pathology and Animal Pathology of the Department of Pathology, A-1090 Vienna, Austria; michaela.schlederer@meduniwien.ac.at (M.S.); lukas.kenner@meduniwien.ac.at (L.K.); 3 Unit of Laboratory Animal Pathology, University of Veterinary Medicine, A-1210 Vienna, Austria; 4 Christian Doppler Laboratory for Applied Metabolomics, A-1090 Vienna, Austria; 5 CBmed GmbH-Center for Biomarker Research in Medicine, A-8010 Graz, Austria

**Keywords:** head and neck cancer, µ-crystallin, thyroid hormones, biomarker, prognosis

## Abstract

Thyroid hormone levels may be associated with disease outcome in head and neck squamous cell carcinoma (HNSCC). µ-Crystallin (CRYM), a thyroid hormone binding protein, blocks intracellular binding of the thyroid hormone T3 to its receptors. In this study, we aimed to analyze the association of CRYM levels with disease outcome in HNSCC patients. We retrospectively assessed immunohistochemical CRYM expression in 121 head and neck cancer patients. Preoperative thyrotropin levels could be extracted for 50 patients. Patients with low thyrotropin levels had a worse prognosis compared to euthyroid patients (5-year overall survival TSH low 20% vs. TSH norm 58%). We observed an association of CRYM+ patients with improved overall survival (5-year overall survival for CRYM+ 78.6% vs. CRYM− 56%). Interaction analysis between CRYM and HPV revealed that this effect was limited to HPV− patients (CRYM+|HPV− HR 0.12, 95% CI 0.01–0.87, *p* = 0.036). These results were replicated in an independent dataset. CRYM expression identified patients with favorable disease progression for HPV− HNSCC patients and could serve as a useful biomarker in this patient population. This study further confirms a correlation of thyroid hormone levels with adverse disease outcome in HNSCC patients, which could be potentially exploited as a therapeutic target.

## 1. Introduction

Head and neck cancer is listed among the ten most frequently diagnosed malignant diseases with an estimated incidence rate of 700,000 cases and 360,000 deaths per year worldwide [1]. The vast majority of head and neck cancers are squamous cell carcinoma (HNSCC) [2]. Survival rates of this cancer entity remain poor and patients are often confronted with disease recurrence despite advances in diagnostic tools and therapeutic possibilities [2]. The underlying biomolecular mechanisms that help to predict treatment response are still in need of further elucidation. So far, the only routinely available protein marker that helps to predict outcome in HNSCC is p16, a surrogate marker for HPV-associated oropharyngeal carcinoma [3]. Therefore, a better comprehension of factors that help to stratify patients into high and low risk for aggressive disease and the identification of new treatment targets is urgently needed. Thyroid hormones may play a significant role in carcinogenesis and disease progression. However, the clinical impact of thyroid hormones in different cancer entities is not uniform [4]. Several in vitro studies investigated the effects of thyroid hormones on different cancer cell lines. The reported results corroborate an ambivalent picture, but, from an overall perspective, studies suggest a potential cancer-stimulating effect of thyroid hormones for some cancer entities [5,6,7]. In HNSCC, Nelson et al. were able to observe an association between patients who developed hypothyroidism after radiation therapy and improved survival in 155 patients [8]. These results could be subsequently verified by a SEER database analysis of nearly 6000 patients [9]. The molecular mechanisms of the carcinogenic role of thyroid hormones in HNSCC are still poorly understood. Physiologically, triiodothyronine (T3) acts genomically through binding to intranuclear receptors, such as thyroid hormone receptor alpha and beta and this complex is able to regulate gene transcription. However, there is also a non-genomic activity via the integrin αVβ3, a membrane protein, or T3-mediated activation of the phosphatidylinositol-3-kinase pathway, a known pro-oncogenic pathway [4,10]. One protein that interferes with the thyroid hormone pathway is µ-crystallin (CRYM). This protein has the capability of NADPH-dependent binding of T3 within the cytoplasm and suppressing T3-mediated gene expression [11,12]. In a recent study by Aksoy et al., CRYM could be identified as a prognostic marker in prostate cancer [13]; however, a potential prognostic or therapeutic relevance of CRYM in HNSCC has not yet been investigated in detail [14]. Taken together, we hypothesize that CRYM expression might be a prognostic factor in HNSCC. Thus, we aimed to investigate the impact of thyroid hormones and CRYM in a HNSCC cohort.

## 2. Materials and Methods

### 2.1. Study Design and Patient Population

The primary study population for the present study was drawn from our in-house HNSCC tissue microarray (TMA) dataset, including 121 patients with histologically confirmed head and neck squamous cell carcinoma. All patients were treated with surgery followed by postoperative radiotherapy at a tertiary center as indicated by the multidisciplinary tumor board between 2002 and 2012. Baseline and outcome data were collected retrospectively from the electronic health record system of our hospital. The last follow-up occurred in September 2019. Exclusion criteria were prior external treatment, secondary primary carcinoma and immunosuppression. 

### 2.2. Assessment of Thyroid Function

Preoperative thyrotropin levels (TSH) were retrospectively extracted from electronic patient health records of our institution and could be included for 50 patients. Blood samples were collected between 2 weeks and 1 day preoperatively. Subclinical hyperthyroidism was defined by the institutional cut-off value at below 0.5 µIU/mL.

### 2.3. Tissue Microarray/Immunohistochemistry

All specimens were examined by a trained pathologist in order to verify the correct histologic entity (squamous cell carcinoma) and determine the boundaries of the primary tumor. In order to construct a TMA, three punch biopsies of 1 mm in diameter and 4 mm in depth were taken from each specimen via a Galileo TMA CK Series-HTS Tissue computer-assisted Microarray Platform (Integrated Systems Engineering Srl., Milan, Italy). 

Subsequently, 2.5 µm-thick sections were cut from the TMA for further immunohistochemical analysis. First, the sections were de-paraffinized and rehydrated following standard protocols. Following antigen retrieval with citrate-buffered saline, 3% H_2_O_2_ was applied in order to inhibit endogenous peroxidase activity. To visualize CRYM expression, the primary antibody (CRYM-Cat# H00001428-M03, clone 6B3, 1:100, Abnova, Taipei City, Taiwan) against the respective protein was applied for 1 h at room temperature. Following the manufacturer’s protocol, primary antibody enhancer was applied for 10 min and afterwards horseradish peroxidase polymer was applied for 15 min. Visualization was achieved by UltraVision Plus Detection System DAB Plus Substrate System (Thermo Scientific, Fremont, CA, USA). Counterstaining was performed with hematoxylin Gill III (Merck, Darmstadt, Germany). The Histoscore was calculated as cytoplasmatic staining intensity (0, no staining; 1, weak; 2, median; 3, strong) multiplied by the percentage of positive stained squamous cell carcinoma cells. Three TMA cores per patient were analyzed and the mean Histoscore was calculated for each patient sample. The samples were categorized into positive (+) and negative (−) CRYM expression at a Histoscore > 10 points by the sample analyst for further statistical analysis. Images were taken on an inverted microscope (Olympus IX73, Olympus Corp., Tokyo, Japan). The sample analyst (L.K-W.) was fully blinded to the clinical dataset. 

### 2.4. Secondary Dataset

The secondary study population was extracted from The Cancer Genome Atlas (TCGA, Firehose Legacy) via cBioportal.org accessed on 25 April 2021 to test our finding using an independent dataset [15,16]. From 530 patients included in the dataset, we excluded observations with missing mRNA expression data, reported overall survival of less than 2 months or incomplete data on cancer staging. Subsequently, we were able to include 443 patients for further analysis. High CRYM was defined as a mRNA z-score threshold > 2, relative to diploid samples. 

### 2.5. Statistical Analysis

Statistical analysis was performed using Stata (Macintosh version 14.0, Stata Corp, Houston, TX, USA). Disease-free survival was defined as the time from surgery to the histologically confirmed occurrence of recurrence and OS was defined as the time from surgery to the time of death from any cause. Categorical variables were reported as absolute counts and percentages, whereas continuous variables were summarized as medians and 25th and 75th percentiles (Q1–Q3). Categorical baseline variables were compared using chi-squared or Fisher’s exact test, and continuous baseline variables were compared using t-test or Wilcoxon rank-sum test, depending on the distribution. OS was estimated using the Kaplan–Meier survivor function, DFS was estimated using the Kaplan–Meier failure function. Log-rank tests were used for the comparison between two groups, and uni- and multivariable Cox proportional hazard models were used for modeling. All multivariable models were adjusted for TNM stage (stages I–II vs. III–IV), smoker status (active smoker vs. nonsmoker) and HPV status (+ vs. −). Pairwise interaction terms between HPV and CRYM were added to evaluate differences in the association with the outcomes and HPV+ and HPV− cases. The proportional hazards assumption was verified by Schoenfeld residuals. In case of separation, Firth correction was applied [17]. Hazard ratios (HR) and 95% CI were calculated. A two-sided *p*-value < 0.05 was considered as statistically significant. 

## 3. Results

### 3.1. Patient Characterisitcs

The baseline characteristics of our cohort are summarized in Table 1. This study included 121 patients from our in-house HNSCC TMA dataset. The age at diagnosis was 59 years (Q1–Q3: 53–63) and 23% of all patients were female (28/121). HPV status was available for 118 patients, of which 25 (21%) were HPV+. The majority of patients were diagnosed with tumors located at the oropharynx (45%), followed by oral cavity (26%), hypopharynx (17%) and larynx (12%). According to the 8th edition AJCC staging, 11 patients (9%) were staged as stage I, 29 (24%) as stage II, 23 (19%) as stage III, 57 (47%) as stage IVA and 1 (1%) as stage IVB. All patients were treated with surgery with curative intent and all patients received postoperative radiotherapy. Adjuvant radiochemotherapy was administered in 19 patients.

### 3.2. Clinical Outcome and Association with TSH Levels and CRYM Status

During a median follow-up of 9.5 years (Q1–Q3: 6.3–12.7), 43 (34%) patients suffered from disease recurrence and 63 (50%) patients died. The estimated 5-year DFS and OS were 66% (95% CI: 55–73) and 59% (95% CI 50–67), respectively. 

**Figure 1:**
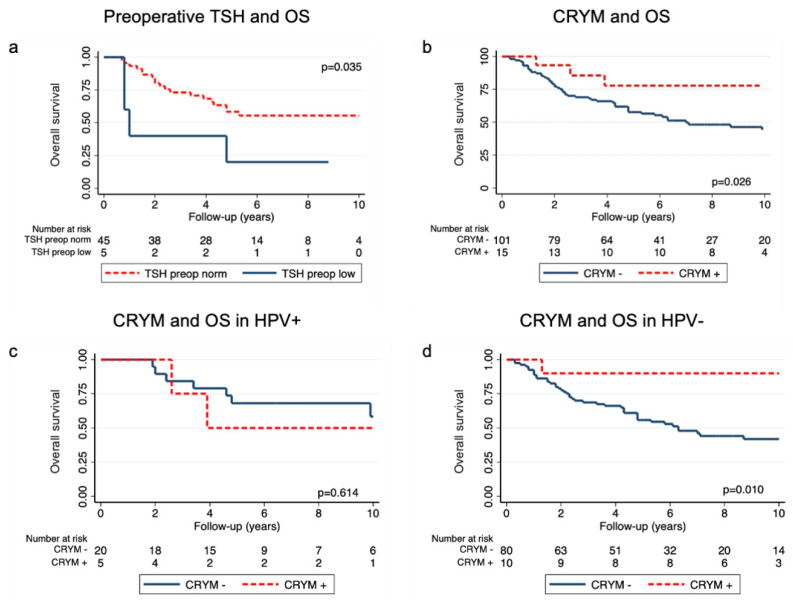
Kaplan–Meier curves for OS according to preoperative TSH levels (**a**), CRYM expression (**b**) and CRYM expression separated for HPV+ (**c**) and HPV− (**d**) patients.

We first explored the association of TSH levels with overall survival. We retrospectively extracted preoperative serum thyrotropin levels from electronic patient health records. Data were available for 50 patients (41%) with a median thyrotropin of 1.16 µIU/mL (Q1–Q3: 0.63–2.08). No patient had thyrotropin greater than 5.5 µIU/mL (cut-off for hypothyroidism) and five patients (10%) had thyrotropin below 0.5 µIU/mL (cut-off for subclinical hyperthyroidism). First, we assessed whether low thyrotropin was associated with overall survival. Survival analysis showed a worse OS for patients with low preoperative TSH levels (5-year OS TSH low 20% (95% CI 1–58) vs. TSH norm 58% (95% CI 42–71), *p* = 0.035, Figure 1a). Univariable Cox models showed a significant association of low TSH with overall survival (HR 2.99, 95% CI 1.01–8.87, *p* = 0.047), but not with DFS (HR 1.54, 95% CI 0.35–6.75, *p* = 0.564, Table 2). Multivariable models were not calculated due to the low number of observed events. 

**Figure 2:**
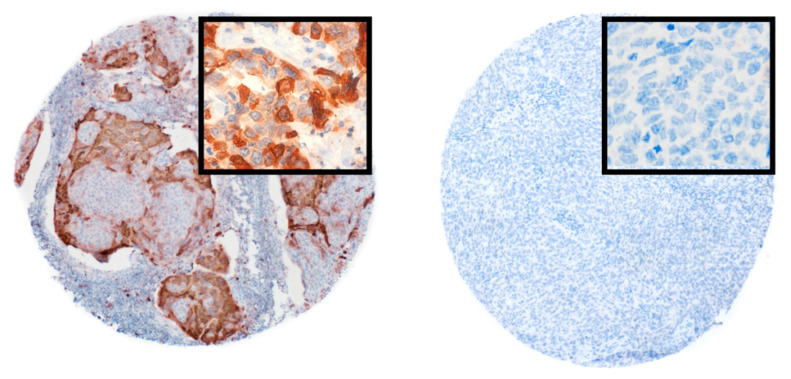
Representative images of immunohistochemical staining of tissue microarrays for CRYM. Positive cytoplasmatic staining (*n* = 15) on the left side and negative staining (*n* = 103) on the right side. Low-power field, 40×; high-power field (insets), 200×.

We next explored the association of CRYM status with clinical outcome of the primary dataset. To determine CRYM levels, we used commercially available antibodies on tissue microarrays using immunohistochemistry. CRYM staining was available for 118 patients and showed a strong positive cytoplasmatic staining in 13% (15/118, Figure 2). Survival analysis revealed a significant difference for OS depending on CRYM status (5-year OS for CRYM+ 78.6% (95% CI 47–93%) vs. CRYM– 56% (95% CI 46–65%, *p* = 0.026, Figure 1b). For DFS, we found a numerically improved DFS; however, this did not reach significance (5-year DFS for CRYM+ 85% (95% CI 51–96%) vs. CRYM− 62% (95% CI 51–71%, *p* = 0.052, Supplementary Materials Figure S1b). Univariable Cox models showed a significant association for CRYM+ patients with improved survival (CRYM+ HR 0.29, 95% CI: 0.09–0.93, *p* = 0.037, Table 2). This result prevailed in a multivariable Cox model after correction for TNM stage, HPV status and smoking status (CRYM+ HR 0.27, 95% CI: 0.08–0.89, *p* = 0.032, Table 2). For DFS, the univariable and multivariable Cox model showed a similar reduction in recurrence risk; however, this did not reach statistical significance (CRYM+ HR: 0.27, 95% CI: 0.06–1.12, *p* = 0.072 and CRYM+ HR: 0.26; 95% CI: 0.06–1.11; *p* = 0.070, respectively, Table 2).

Next, we analyzed associations between thyrotropin levels and CRYM status. No differences for thyrotropin levels depending on CRYM status were found (CRYM+ thyrotropin = 1.56 µIU/mL (95% CI = 1.18–1.93) vs. CRYM- thyrotropin = 1.1 µIU/mL (95% CI 0.64–1.55), *p* = 0.289, Supplementary Materials Figure S1a). Furthermore, low TSH levels were not associated with CRYM status (*p* = 0.401; Supplementary Materials Table S2). Finally, we aimed at analyzing whether CRYM+ would abrogate the effect of low preoperative TSH on OS. However, an interaction analysis between CRYM and TSH could not be obtained due to no observation of patients classified as CRYM+ and TSH low. No significant associations of CRYM status were found with other clinicopathological characteristics (Supplementary Materials Table S2). 

Finally, because of the fundamentally different tumor biology of HPV-associated HNSCC, we performed a subgroup analysis for CRYM and HPV status. We observed a difference for OS depending on HPV status of CRYM+ patients. CRYM expression had only a protective effect on survival in HPV− patients (5-year OS CRYM−|HPV− 55% (95% CI 44–66) vs. CRYM+|HPV− 89% (95% CI 45–98), *p* = 0.010, Figure 1c, d). Univariable Cox models for OS including a pairwise interaction of CRYM with HPV status revealed a significant interaction (CRYM+|HPV− HR 0.12, 95% CI 0.01–0.87, *p* = 0.036, Table 2). In multivariable analysis, these results remained unchanged after correction for possible confounders (HR: 0.11, 95% CI 0.01–0.84, *p* = 0.033, Table 2).

### 3.3. Validation of Our Findings in an Independent Dataset

**Figure 3:**
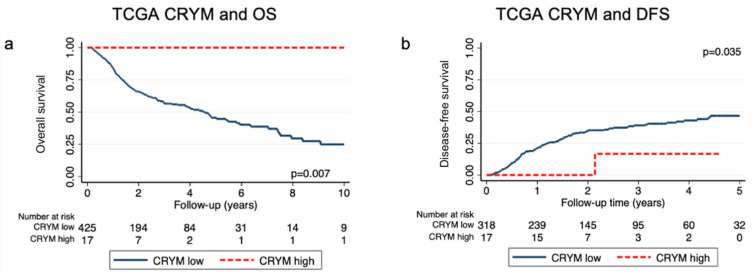
Kaplan–Meier estimator for OS (**a**) and KM failure function for DFS (**b**) of the secondary dataset.

To test our findings in an independent dataset, we assessed CRYM mRNA levels in the TCGA dataset “Firehose Legacy” and its association with survival. A total of 443 patients could be included. Baseline characteristics of the TCGA dataset are summarized in Table 1, right column. One hundred and eighty-seven patients (42%) had died with a 5-year OS of 48% (95% CI: 43–54) and 124 patients (37%) suffered from disease recurrence with a 5-year DFS of 51% (95% CI: 45–57). The median follow-up was 2.9 years (Q1–Q3: 1.76–4.71). Patients were stratified for CRYM mRNA levels based on an empirical cut-off at a z-score above 2 standard deviations (= CRYM high). A total of 17 patients (4%) had high CRYM mRNA levels. The 5-year OS and DFS of those patients were significantly improved compared to CRYM-low patients (5-year OS CRYM high: 100% (95% CI: NA) vs. CRYM low: 47% (95% CI: 41–52), *p* = 0.007 and 5-year DFS CRYM high 89% (95% CI: 43–98) vs. CRYM low 50% (95% CI: 43–56), *p* = 0.035, Figure 3). Univariable Cox models showed statistically significant differences for overall survival (CRYM high HR: 0.08; 95% CI: 0.01–0.54, *p* = 0.002) and DFS (CRYM high HR: 0.26; 95% CI: 0.03–0.95; *p* = 0.04, Supplementary Materials Table S1). After correction for possible confounders, the significance of high CRYM expression on OS prevailed (HR: 0.08; 95% CI: 0.01–0.53; *p* = 0.002) while the effect on DFS was no longer significant (HR: 0.30; 95% CI: 0.03–1.09; *p* = 0.072, Supplementary Materials Table S1). 

Finally, we replicated the subgroup analysis separated for HPV status. We found the effect on OS only to be significant in the HPV− subgroup (5-year OS HPV+ CRYM high vs. low not evaluable vs. 57% (95% CI 38–72), *p* = 0.313 and HPV− CRYM high vs. low 100% (95% CI NE) vs. 45% (95% CI 39–51, *p* = 0.019)). Cox models including an interaction term between CRYM and HPV were not obtained due to the low number of events in the CRYM high|HPV+ group.

## 4. Discussion

In this retrospective observational cohort study, we demonstrated that patients with subclinical hyperthyroidism had significantly impaired survival compared to euthyroid patients. We furthermore showed, for the first time, that CRYM expression was associated with increased survival in HNSCC. When analyzing the interaction between CRYM and HPV status, we found that this effect was localized to HPV− patients. Furthermore, we demonstrated the specificity of this finding by excluding associations with any other clinicopathological characteristics. Finally, we externally validated this finding in silico at the mRNA level in a cohort of The Cancer Genome Atlas. 

Previous studies on the effect of thyroid hormones and disease outcome in HNSCC found that hypothyroidism was associated with improved survival and increased disease-free survival [8,9]. In our cohort, however, no patient reached the cut-off for hypothyroidism used in the study by Nelson et al. (>5.5 µIU/mL). We therefore assessed the effect of subclinical hyperthyroidism (TSH cut-off at <0.5 µU/mL) on disease outcome. We found that patients with subclinical hyperthyroidism were three times more likely to die, compared to euthyroid patients in univariable Cox models. Multivariable models were not feasible because of the low number of observed events based on the recommendation by Heinze et al. [18]. Notably, the literature on the association of subclinical hyperthyroidism and outcome in cancer patients is sparse. Hellevik et al. found an association of overall increased cancer risk with subclinical hyperthyroidism at a cut-off < 0.5 mIU/L in a population-wide study of over 29,000 participants [19]. Muller et al. investigated the effect of thyroid function on the survival of patients with early breast cancer in a large-scale study and found no effect on disease outcome. However, blood samples in this study were taken over a year after surgery [20]. 

To the best of our knowledge, no other studies have investigated the effect of subclinical hyperthyroidism on disease outcome in cancer patients to date. 

Next, we hypothesized that CRYM expression might positively influence disease outcome by blocking thyroid hormones and would therefore harbor prognostic information. In our cohort, a rather small proportion of 13% stained positive for CRYM. We found that those patients had a significant relative risk reduction of over 70% for OS. Furthermore, the recurrence risk was also similarly reduced in CRYM+ patients. Although this result was borderline significant in our cohort, based on the hazard ratio and confidence intervals it appeared to be a potentially relevant prognostic marker [21]. Interestingly, in a subsequent interaction analysis with HPV status, we found that this effect was limited to HPV– patients. The combination of CRYM+ with HPV− status led to a relative risk reduction of almost 90%. For DFS, the results for an interaction with HPV also showed a stronger association with HPV− patients compared to HPV+ patients, although this did not reach statistical significance. Notably, the borderline significant results remained unchanged for HPV− patients after correction for other confounders in a multivariable model. Hence, it appeared that this effect might also be clinically relevant and would potentially reach statistical significance in a larger cohort. The biological mechanisms behind an association of HPV with thyroid hormones are largely unexplored to date. The HPV oncoproteins E6 and E7 alter the function of cellular regulatory proteins, but have furthermore been shown to directly interact with nuclear receptors, such as the thyroid hormone receptors, as well [22]. Direct activation of nuclear thyroid hormone receptors by HPV oncoproteins could potentially explain an escape mechanism of blocked thyroid hormone signaling by CRYM. Notably, this is purely speculative and it is worth mentioning that the molecular pathways for HPV infection and thyroid hormone signaling are complex and potentially interact at multiple pathways [23,24,25].

Finally, we aimed to validate our findings in an independent dataset. We utilized a dataset from the TCGA, namely “Firehose Legacy”, to replicate our analysis at the mRNA level. In the secondary dataset, an even smaller proportion of 4% was categorized as CRYM high at an empirical cut-off at a z-score > 2. The difference between this result and the 13% positive rate for CRYM staining in the primary data set could be due to processes beyond mRNA concentrations, such as translation rates or modulations and protein half-life or synthesis delay [26]. Here, we also observed an excellent OS for CRYM-high patients with approximately 90% relative risk reduction. The secondary dataset also corroborated our findings for DFS, with a 70% relative risk reduction for disease recurrence in CRYM-high patients. The subsequent subgroup survival analysis separated for HPV status supports our finding that the prognostic effect of CRYM might be associated with HPV status. Taken together, the results of the secondary dataset corroborate our findings on the prognostic effect of CRYM in HNSCC. 

A main limitation of this study that needs to be discussed is the relatively small cohort, especially the low number of patients with CRYM positive staining and patients with preoperative hyperthyroidism. An interaction analysis between CRYM and TSH, for example, could not be calculated due to no observations in the CRYM+|TSH low group. Additionally, some associations which were not significant at a significance level of *p* ≤ 0.05 might have crossed this threshold in a larger cohort. Secondly, CRYM expression was evaluated in TMA samples rather than whole tissue sections, therefore potentially neglecting the heterogenous distribution of CRYM expression. We aimed to limit this potential bias by constructing the TMA including three random cores per specimen. Thirdly, although we could validate our findings at the mRNA level of an independent dataset, the results need to be validated at the protein level. Particularly the observed interaction with HPV status was an exploratory finding and needs to be validated externally. Lastly, because of the retrospective design of the study, selection bias cannot be fully excluded.

In conclusion, our data indicate that CRYM might influence the disease outcome of HPV− HNSCC patients, and could serve as an easily applicable biomarker in the clinic. Furthermore, our findings on the effect of subclinical hyperthyroidism build on the existing literature and corroborate the relevance of thyroid hormones on disease outcome in HNSCC. In combination with our data, it seems crucial to routinely monitor thyroid hormone levels in the HNSCC patient population. This finding could also have a therapeutic significance, as T3 synthesis can be lowered with methimazole and thus hypothyroidism could be induced. The possibility of adjusting thyroid hormone levels to improve prognosis would need to be evaluated in a timely manner in preclinical and clinical studies. 

## 5. Conclusions

CRYM expression could serve as a useful biomarker for the identification of HPV− HNSCC patients with a favorable prognosis. This study further corroborates a correlation of thyroid hormone levels with adverse disease outcome in HNSCC patients.

## Figures and Tables

**Table 1:** Baseline characteristics of the study population for the primary and secondary dataset. NA, not available.

	TMA	TCGA
	(*n* = 121)	(*n* = 443)
**Gender**		
Female	28 (23%)	119 (27%)
Male	93 (77%)	324 (73%)
**Age at diagnosis**	59	61
(Q1–Q3)	(53–63)	(53–68)
**T-stage**		
T1	24 (20%)	47 (11%)
T2	63 (52%)	132 (30%)
T3	21 (17%)	96 (22%)
T4		10 (2%)
T4A	13 (11%)	154 (35%)
T4B		4 (1%)
**N-stage**		
N0	23 (25%)	169 (38%)
N1	23 (25%)	66 (15%)
N2	-	11 (2%)
N2a	7 (8%)	8 (2%)
N2b	31 (33%)	95 (21%)
N2c	8 (9%)	48 (11%)
N3	1 (1%)	8 (2%)
N0 (HPV+)	2 (8%)	-
N1 (HPV+)	5 (20%)	-
N2 (HPV+)	16 (64%)	-
N3 (HPV+)	2 (8%)	-
NX	-	38 (9%)
**TNM staging**		*n* = 435
I–II	40 (33%)	100 (23%)
III–IV	81 (67%)	335 (77%)
**HPV**	*n* = 118	*n* = 421
+	25 (21%)	49 (12%)
−	93 (79%)	372 (88%)
**Primum**		
Hypopharynx	21 (17%)	8 (2%)
Larynx	14 (12%)	100 (235)
Oral cavity	32 (26%)	284 (64%)
Oropharynx	54 (45%)	49 (11%)
**Alcohol use**	*n* = 106	NA
Non-drinker	65 (61%)	
Active drinker	41 (39%)	
**Smoking status**	*n* = 120	
Non/ex-smoker	38 (32%)	274 (64%)
Smoker	82 (68%)	154 (36%)
**Marker expression**	*n* = 118	
CRYM +	15 (13%)	17 (4%)
**TSH**	*n* = 50	NA
Low	5 (10%)	

**Table 2:** Uni- and multivariable time-to-event analysis for OS and DFS.

	Univariable			Multivariable		
	HR	95% CI	*p*-Value	HR	95% CI	*p*-Value
**Overall survival**						
TSH low	2.99	1.01–8.87	0.047	-	-	-
CRYM+	0.29	0.09–0.93	0.037	0.27	0.08–0.89	0.032
HPV+	0.62	0.15–2.60	0.522	10.7	0.84–137.6	0.067
HPV−	0.12	0.01–0.87	0.036	0.11	0.01–0.84	0.033
**Disease-free survival**						
TSH low	1.54	0.35–6.75	0.564	-	-	-
CRYM+	0.27	0.06–1.12	0.072	0.26	0.06–1.11	0.070
HPV+	0.39	0.05–2.94	0.368	0.53	0.06–4.25	0.554
HPV−	0.18	0.02–1.37	0.100	0.18	0.02–1.40	0.104

Multivariable models were adjusted for TNM stage, smoker status and HPV status. For HPV, we calculated a pairwise interaction with CRYM; therefore, separate hazard ratios for HPV+ and HPV− patients are reported.

## Data Availability

The datasets generated and analyzed during the current study are available from the corresponding author on reasonable request.

## Supplementary Materials

The following are available online at https://www.mdpi.com/article/10.3390/jpm11121330/s1, Figure S1: Kaplan–Meier failure functions for the primary dataset, Table S1: Uni- and multivariable time-to-event analysis of the secondary dataset, Table S2: Characteristics of CRYM expression and clinic-demographic data.
