# Supplementary material for: µ-Crystallin Is Associated with Disease Outcome in Head and Neck Squamous Cell Carcinoma
Source: J Pers Med. 2021 Dec 8;11(12):1330. doi: 10.3390/jpm11121330 (PMC8703347; doi:10.3390/jpm11121330)

Table S1 Uni- and multivariable time-to-event analysis for OS and DFS of the secondary dataset. Multivariable models were adjusted for TNM-stage, smoker status and HPV status.

	**Univariable**			**Multivariable**		
	HR	95% CI	p-value	HR	95% CI	p-value
**Overal survival**						
CRYM +	0.08	0.00-0.53	0.002	0.08	0.00-0.52	0.002
**Disease-free survival**						
CRYM +	0.26	0.03-0.95	0.040	0.30	0.03-1.09	0.072
Multivariable models were adjusted for TNM-stage and HPV status						

Table S2 Characteristics of CRYM expression, clinical and demographic data of the primary dataset.

	CRYM	
	-	+
Sex<br>Male<br>Female	 77 (87%)<br>24 (89%)	 12 (13%)<br>3 (11%)
	p=0.748	
Age (mean)<br>(Q1-Q3)	57.5 (53-63)	56.9 (48-64)
	p=0.800	
T-classification<br>T1-T2<br>T3-T4	 72 (86%)<br>29 (91%)	 12 (14%)<br>3 (9%)
	p=0.481	
N-classification<br>N0<br>N+	 25 (96%)<br>76 (84%)	 1 (4%)<br>14 (16%)
	p=0.185	
Staging<br>I-II<br>III-IV	 34 (87%)<br>67 (87%)	 5 (13%)<br>10 (13%)
	p=0.980	
HPV Status<br>HPV HR –<br>HPV HR +	 79 (89%)<br>19 (79%)	 10 (11%)<br>5 (21%)
	p=0.219	
Alcohol use<br>Non-drinker<br>Active drinker	 55 (89%)<br>33 (82.5%)	 7 (11%)<br>7 (17.5%)
	p=0.374	
Smoking status<br>Non-Smoker<br>Smoker	 29 (81%)<br>72 (90%)	 7 (19%)<br>8 (10%)
	p=0.161	
TSH low	5 (100%)	0 (0%)
	p=0.401	

Figure S1 Tukey boxplot for preoperative TSH levels separated by CRYM status (a). Kaplan Meier failure function for DFS of the primary dataset (b) and separated for HPV status (b,c)

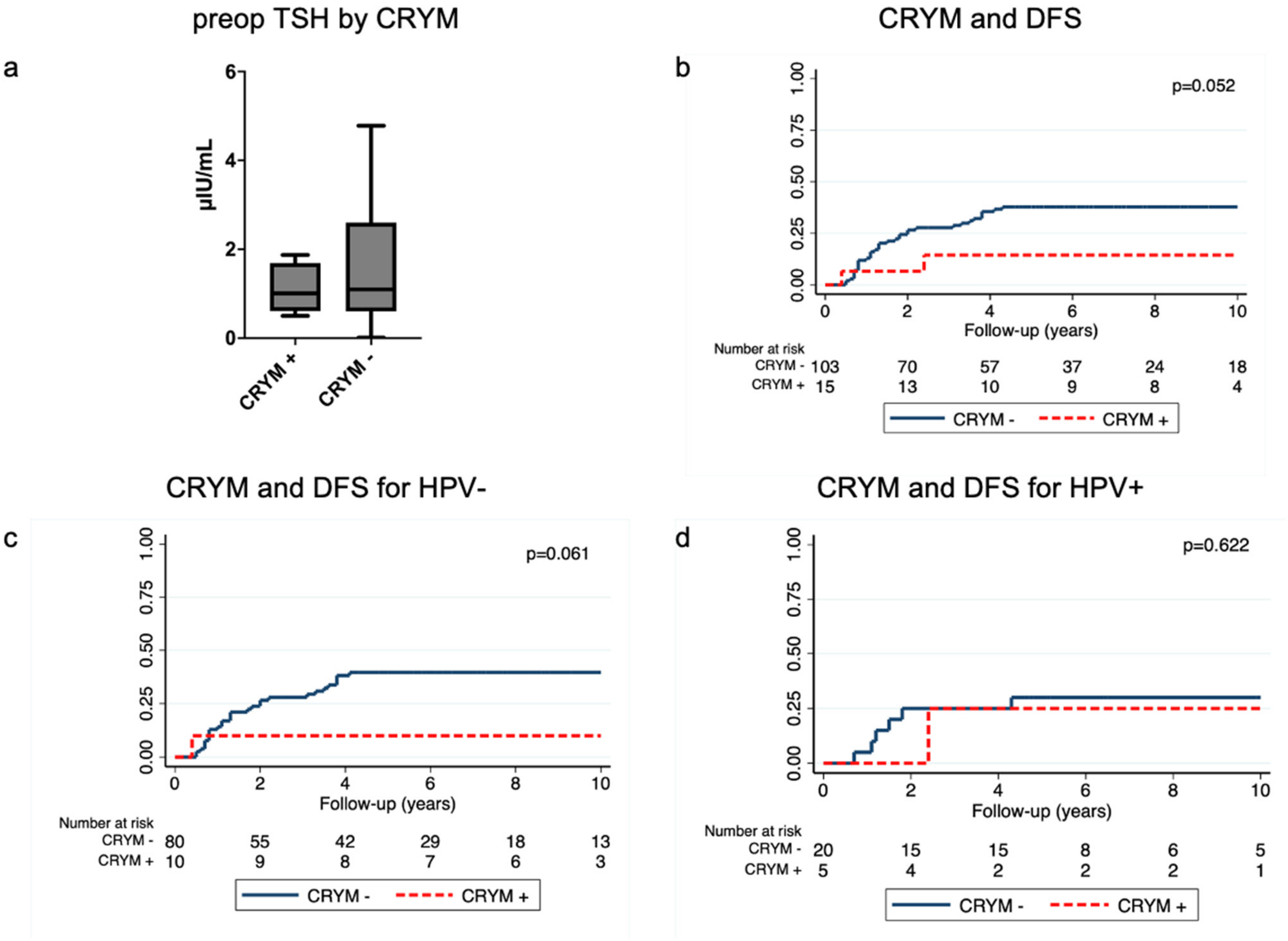

Supplement: Supplementary file 1 [file jpm-11-01330-s001.zip › jpm-1469851-supplementary.pdf]
